# Antimicrobial and Antioxidant Activities and Phytochemical Analysis of *Rosmarinus officinalis* L. Pod and *Thymus vulgaris* L. Leaf Ethanolic Extracts on *Escherichia coli* Urinary Isolates

**DOI:** 10.1155/2023/4171547

**Published:** 2023-04-25

**Authors:** Amel Ahmed Alrasheed, Ayat Ahmed Alrasheid, Wafaa Mohamed Abdalla, Samar Mohammed Saeed, Hind Haidar Ahmed

**Affiliations:** ^1^Department of Microbiology, Faculty of Medical Laboratory Science, Sudan University of Science and Technology, Khartoum, Sudan; ^2^Department of Pharmacognosy, Faculty of Pharmacy, University of Medical Sciences and Technology, Khartoum, Sudan

## Abstract

The indiscriminate use of antibacterial agents has resulted in one of the largest recent global health problems, which is the emergence of bacterial resistance. This study aimed to examine the antimicrobial and antioxidant activities of ethanolic extracts of the two medicinal plants; *Rosmarinus officinalis* pods and *Thymus vulgaris* leaves on *Escherichia coli* urinary isolates. Both plants were extracted by absolute ethanol, and various concentrations (100, 50, 25, and 12.5 mg/ml) of the ethanolic extracts were prepared and tested against 53 urinary isolates of *E*. *coli*. An antibiotic susceptibility test was performed using chloramphenicol, gentamycin, amoxicillin, ceftriaxone, and ciprofloxacin against isolated bacteria. The antioxidant activity was measured using the DPPH method. The chemical analysis of both extracts was determined using gas chromatography-mass spectrometry (GC/MS) technique. The results showed that 88.7% of the isolated bacteria were sensitive to chloramphenicol and 87% were sensitive to gentamycin, while all isolates were resistant to amoxicillin, 13% of *E*. *coli* isolates were found to be multidrug-resistant (MDR). The inhibitory zone of *R. officinalis* extract against *E*. *coli* ranged between 8 and 23 mm and for *T*. *vulgaris* extract ranged between 8 and 20 mm at concentrations between 25, 50, and 100 mg/ml. The MIC of both extracts against isolates is between 12.5 and 50 mg/ml, while the MBC is between 50 and 100 mg/ml. The DPPH radical scavenging potential of *T. vulgaris* was 83.09%, followed by *R. officinalis* (81.26%). The chemical analysis by GC-MS of *R. officinalis* showed that the most active compounds were: eucalyptol (18.57%), bicycloheptan (10.01%), and octahydrodibenz anthracene (7.44%) and for *T. vulgaris* the most active compounds were: thymol (5.7%), phytol (7.92%), and hexadecanoic acid (18.51%). *R. officinalis* and *T. vulgaris* ethanolic extracts possessed antimicrobial and antioxidant activities and were found to be rich natural sources of active constituents used as traditional medicine.

## 1. Introduction

The excessive use of antibiotics contributed to the emergence and spread of antibiotic-resistant bacteria in Sudan. *Escherichia coli* remain one of the most frequent causes of several common bacterial infections in humans. It is a type of Gram-negative bacterium and has been documented to be the most common pathogen associated with urinary tract infections in many countries, causing both community and hospital-acquired urinary tract infections (UTI); it is among the most common infections with an increasing resistance to antimicrobial agents [1]. For centuries, plants have been used for a wide variety of purposes, including the treatment of infectious diseases [2]. Aromatic plants and spices also have great importance for the pharmaceutical industry, cosmetics, and food. According to the World Health Organization, approximately 80% of the world's population, mostly in developing countries, still relies on medicinal plants for primary health care [3]. Many plants have been studied because of their bioactive properties and great antioxidant potential. Antioxidants reduce oxidative stress in cells and have therefore become useful in the treatment of many human diseases, including cancer, cardiovascular, and inflammatory diseases [4]. Synthetic drugs are widely used against microorganisms; unfortunately, they develop resistance to many antibiotics as a result of their indiscriminate use. Furthermore, these antibiotics sometimes cause allergic reactions and immune suppression. The use of plants is safer for human health and for the environment. *Rosmarinus officinalis*, which belongs to the Lamiaceae family, is a plant with green, picked, and fragrant leaves and pods, also rich in chemical compositions that possess antimicrobial and antioxidant properties as reported in various researches [5]. *Thymus vulgaris* which belongs to the Lamiaceae family too, has been widely used for its organoleptic and medicinal properties, and they possess anti-inflammatory, antioxidant, antibacterial, and antifungal activities [6].

## 2. Materials and Methods

### 2.1. Preparation and Extraction of Plant Materials

The commercial plant materials were obtained from the local market in 2019 and authenticated in the Medicinal and Aromatic Plants and Traditional Medicine Research Institute. Herbal samples were purchased and washed, then air-dried at room temperature (30°C) for approximately 5 days. After that, each sample was ground to a coarse powder using a pestle and mortar and stored in a clean container, ready for extraction. About seventy grams of each plant were weighted, macerated in absolute ethanol, and left at room temperature for three days. Extracts were filtered through filter paper and vacuum concentrated at 80°C [5].

### 2.2. Bacteria Strain

The bacterial isolates were provided by a Police's Hospital, inoculated on nutrient slope and incubated aerobically at 37°C for 24 hours, preserved at 20°C and re-identified by morphological colony identification and biochemical tests to confirm the *E. coli* organisms.

### 2.3. Preparation of Bacterial Suspension

Stock cultures were maintained at 4°C on the nutrient agar. Active cultures for experiments were prepared by transferring a loopful of bacterial cells from the stock cultures to a tube containing 1 ml of normal saline for bacteria. The cultures were incubated for 24 hours at 37°C. The cultures were then diluted with normal saline to achieve the McFarland 0.5 turbidity standard [7].

### 2.4. Antimicrobial Activity (Disc Diffusion Method)

Twenty ml aliquots of Mueller Hinton agar were adjusted into sterile Petri dishes. The isolates and standardized bacterial stock suspension were adjusted to 0.5 McFarland and streaked on Mueller Hinton agar medium plates using a sterile cotton swab. Sterile filter paper discs (6 mm Whatman No. 1) were soaked with different concentrations (12.5, 25, 50, and 100 mg/ml) of the extracts, and then placed on the surface of the agar. The plates were incubated for 24 hours at 37°C and the diameters of the inhibition zones were measured in mm [8]. MIC was determined by the lowest concentration that inhibited the growth of *E. coli* [8]. This experimental study was conducted at the Microbiology Research Laboratory, Sudan University of Science and Technology.

### 2.5. Susceptibility Test of Isolates against Selected Antimicrobial Discs

Disc diffusion method was used to determine the antibiotic susceptibility of *E. coli.* The isolates were suspended in sterile normal saline, and the turbidity was adjusted to a standard concentration of 0.5 McFarland solutions. The isolates were then inoculated on Mueller Hinton agar. The following antibiotic discs were used in this study: chloramphenicol (30 mcg), gentamicin (10 mcg), amoxicillin (25 mcg), ceftriaxone (30 mcg), and ciprofloxacin (50 mcg). All discs contained a precise concentration of the antibiotics that were individually placed 1 cm from the wall and from each other. The plates were then incubated at 37°C for 24 hours. The diameter zones of inhibition were measured in millimeters and interpreted according to the Clinical Laboratory Standard Institute (CLSI) protocol [9].

### 2.6. Broth Dilution Technique for Detection of MIC and MBC

The minimum inhibitory concentration (MIC) and minimum bactericidal concentration (MBC) of both extracts were determined using standard procedures. 100 mg of each extract was dissolved in 1 ml of dimethyl sulfoxide (DMSO). Serial dilutions at concentrations of 50, 25, and 12.5 were prepared. Bacterial concentration was calibrated to a McFarland standard of 0.5. The bacteria were added to the diluted extract. The turbidity of the solution in each tube was observed after 24 hours in order to find out if there was any bacterial growth. The tubes that showed no turbidity were recorded as MIC values. The MBC value was considered as the lowest concentration of the extract dilution showing no visible growth [10].

### 2.7. Determination of Antioxidant Activity by DPPH Method

The DPPH radical scavenging was determined according to the method of Hilmi et al. [11] with some modifications. In a 96-well plate, the test samples were allowed to react with 2.2 Di (4-tetra-octylphenyl)-1-picryl-hydrazyl (DPPH) for half an hour at 37°C. The tested samples were dissolved in DMSO while DPPH was prepared in ethanol. After incubation, absorbance was measured at 517 nm using multiple reader spectrophotometers. The percentage of radical scavenging activity was determined. Ascorbic acid was used as a standard and all tests and analyses were run in triplicates [12].

### 2.8. Gas Chromatography-Mass Spectrometry

The qualitative and quantitative analyses of the samples were carried out by using GC-MS technique model (GC-MS-QP2010-Ultra) from Japan Shimadzu Company, with a capillary column (Rtx-5 ms–30 m × 0.25 mm × 0.25 *μ*m). Each sample was injected by using the split mode, with helium as the carrier gas passed with a flow rate of 1.61 ml/min. The temperature program was started from 60°C with a rate of 10°C/min to 300°C as the final temperature degree. Each sample was analysed by using scan mode in the range of *m*/*z* 40–500 charges to ratio, and the total run time was 26 min. Identification of components for the samples was achieved by comparing their retention times and mass fragmentation patterns with those available in the library from the National Institute of Standards and Technology (NIST).

### 2.9. Data Analysis

The data were analysed by using Statistical Package for Social Sciences (SPSS v.20) program to get the mean and the standard deviation.

## 3. Results

### 3.1. Antimicrobial Activity

Antibiotics susceptibility test was applied against 53 *E*. *coli* urinary isolates. The 47 (88.7%) isolates were sensitive to chloramphenicol, 46 (87%) sensitive to gentamycin, no isolate (0%) was sensitive to amoxycillin, and there were 7 (13%) *E. coli* isolates found to be multidrug-resistant, as shown in Table 1.

The antibacterial activity at concentrations of 12.5, 25, 50, and 100 mg/ml of the ethanolic extracts of *R. officinalis* pod and *T. vulgaris* leaves were determined against 53 urinary isolates of *E. coli* using the disc diffusion method. Both extracts showed variable activity against the tested bacteria. The inhibition zone of *R. officinalis* against *E. coli* ranged between 8 and 23 mm and for *T. vulgaris* between 8 and 20 mm, as shown in Tables 2 and 3. The MIC of both extracts against multidrug-resistant* E. coli* isolates ranged between 12.5 and 50 mg/ml, while the MBC ranged between 50 and 100 mg/ml, as shown in Table 4.

### 3.2. Antioxidant Activity

The highest DPPH radical scavenging potential showed in *T. vulgaris*, it was found to be 83.09%, followed by *R. officinalis* (81.26%), as shown in Table 5.

### 3.3. GC-MS Analysis

**Figure 1:**
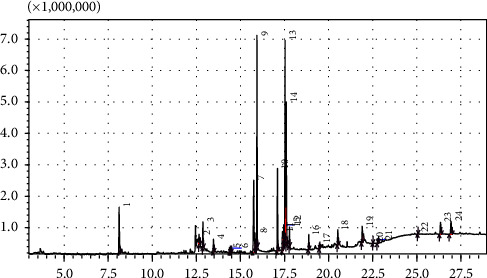
GC/MS chromatogram of ethanolic extract of *T*. *vulgaris*.

The GC/MS analysis of both plants revealed the presence of different chemical constituents which have different biological activities. The chemical analysis of *R*. *officinalis* revealed the presence of 77 compounds, the most abundant compounds were; eucalyptol, bicycloheptan, and octahydrodibenz anthracene. The analysis of *T*. *vulgaris* (Figure 1) revealed the presence of 24 compounds, the most abundant compounds were: thymol, phytol, and hexadecanoic acid. The concentrations and biological activities of the compounds found in the extracts are shown in Tables 6 and 7.

## 4. Discussion

Medicinal plants contain components that have different therapeutic potential; they have been utilized as treatments for human infections for centuries [25]. Urinary tract infections (UTIs) are the second most prevalent cause for hospital visits and one of the most common causes of morbidity in the general population [26]. Antibiotic resistance against bacterial pathogens associated with urinary tract infections (UTI) is rapidly increasing worldwide [27]. *E. coli* is one of the most common causes of urinary tract infections [28]. In this study; 7 (13%) *E. coli* urinary isolates showed multidrug resistance which is in an agreement with Saeed et al. [29] who found that *E. coli* urinary isolate in Sudan was highly resistant to the most utilized antibiotics, it showed highly resistant rates against ampicillin, amikacin, amoxycillin, nitrofurantoin, co-trimoxazole, tetracycline, and cephalosporins that have continued to increase in the past decade and now approaches 40%, also *E. coli* showed high susceptible rates to gentamicin.

*R. officinalis* extract at a concentration 100 mg/ml showed an inhibition zone of 12 mm against *E. coli*, which disagreed with a study done by Jiffri and Zahira [27] in Iran, who reported that a zone of inhibition of leaf extract was found to be 10.5 mm at a concentration of 500 mg/ml. The differences in results may be due to the difference in bacterial cell wall, origin of the *R. officinalis*, or the difference in part used.

Antimicrobial activity with the MIC of *R. officinalis* ethanolic extract against *E. coli* was examined using the concentrations 100, 50, 25, and 12.5 mg/ml. The MIC for one isolate was 12.5 mg/dl, and other isolates ranged from 25 mg/dl and above; the results were mismatched with Genena et al. [30] in Brazil, who reported that a higher concentration of the extract needed to inhibit *E. coli* was 320 mg/ml, also disagreed with Golshani and Shaifzadeh [5]. Another study in Iran, reported that the MIC of the *R. officinalis* L. extract that inhibits *E. coli* growth was 200 mg/ml. The differences may be due to cell membrane permeability or due to other genetic factors.

*T. vulgaris* extract showed a zone of inhibition of 11 mm against *E. coli* at 100 mg/ml. These findings were differed from those obtained by Mohsenipour and Hassanshahian [31] who reported that the ethanolic extract of *T. vulgaris* was not effective against *E. coli*; discrepancy may be due to the resistance of *E. coli* strains.

MIC of *T. vulgaris* ethanolic extract against *E. coli* was examined using the concentrations 100, 50, 25, and 12.5 mg/ml. It was found that MIC ranged from 12.5 mg/dl and above. These results were conflicted with Jiffri and Zahira [27] in Iran, who reported that a higher concentration of the extract needed to inhibit *E. coli* was 500 mg/ml. Also varied from Bayoub et al. [19] in Morocco who found MIC of the *T. vulgaris* extract that inhibit *E. coli* growth was 1.56 mg/ml. The differences might be due to use of other method or resistance of *E. coli* due to cell membrane permeability, or other genetic factors.

The present result showed the DPPH radical scavenging activity of *R. officinalis* L. was 81.26% and it was comparable with the findings of Martínez et al. [32] in Spain whom reported that rosemary exhibited a high radical scavenging activity, also, another study by Kumuda et al. [12] in India who found the DPPH radical scavenging activity of *R. officinalis* L. was 77.37%.

The present result showed the DPPH radical scavenging activity of *T. vulgaris* L. was 94.51% and it was similar with the findings of Wisam et al. [33] in Pakistan,who reported that thyme exhibited a high radical scavenging activity, and Iuliana et al. [34] in Romania who found that *T. vulgaris* L. presents significant antioxidant activity.

In this study, the analysis of *R. officinalis* L. extract by GC-MS led to the identification of 77 compounds by comparison of recorded mass spectra with those of a computer library but the most active compounds were eucalyptol, camphor, endo-borneol, beta-Amyrin, wogonin, beta-Amyrone, and octahydrodibenz [*a*, *h*] anthracene. Eucalyptol was obtained at the highest percentage was considered as antimicrobial agent and camphor which was found in the extract has a great antioxidant and antimicrobial activity. These results were matched with Rashid et al. [8] in Baghdad who found that rosemary extracts contain endo-borneol, camphor, and eucalyptol but at different concentrations, this variation may be due to seasonal variation, plant status, and the extraction method.

The analysis of *T. vulgaris* extract by GC-MS led to the identification of 24 compounds by comparison of recorded mass spectra with those of a computer library but the most active compounds were thymol, phytol, hexadecanoic acid- ethyl ester, stigmasterol, linolelaidic acid ethyl ester, octadecatrienoate, and gamma sitosterol. Hexadecanoic acid, ethyl ester was obtained at the highest percentage was considered as antioxidant. Hexadecanoic acid and thymol, which were found in the extract, possess antimicrobial activity. These results were agreed with Iuliana et al. [34] in Romania who found *T. vulgaris* extract contains thymol, camphor, palmitic, stearic, oleic, and linoleic and linolenic acids but at different concentrations. The differences may be due to seasonal variation, plant condition, or the extraction methods.

## 5. Conclusion

Screening of *R. officinalis* and *T. vulgaris* against *E. coli* as well as the radical scavenging potential showed that the ethanolic extracts of the two plants have broad antimicrobial and antioxidant activities. Both extracts have antibacterial activity which might justify the use of those herbs in the pharmaceutical industries for the production of new semi‐synthetic agents against *E. coli*. Chemical analysis showed that some compounds detected from the two plants were found to be antimicrobial agents. This study demonstrated support for the claimed uses of the plants in the traditional medicine. The results of the present study gave solid grounds that both plants extracts possess a medicinal potential to develop new phytopharmaceutical drugs, further research studies are warranted to isolate the active components.

## Figures and Tables

**Table 1:** Antibiotics susceptibility test against *E*. *coli* urinary isolates.

Antibiotic (mcg)	Antimicrobial susceptibility pattern		
	Sensitive (%)	Intermediately sensitive (%)	Resistant (%)
Chloramphenicol (30)	47 (88.7)	0 (0)	6 (11.3)
Ceftriaxone (30)	20 (39.6)	10 (18.9)	22 (41.5)
Gentamicin (10)	46 (87)	0 (0)	7 (13)
Ciprofloxacin (50)	38 (71.7)	0 (0)	15 (28.3)
Amoxycillin (25)	0 (0)	0 (0)	53 (100)

**Table 2:** Antibacterial activity of different concentrations of ethanolic extract of *R. officinalis* against *E. coli* urinary isolates.

Concentrations (mg/ml)	Inhibition zone minimum–maximum (mean ± SD) in (mm)	Number of isolates showed inhibition zone (%)
12.5	0–8 (4 ± 4)	1 (2)
25	8–12 (10 ± 2)	18 (34)
50	8–19 (13.5 ± 6)	34 (64.2)
100	9–23 (16 ± 7)	48 (90.6)

**Table 3:** Antibacterial activity of different concentrations of ethanolic extract of *T*. *vulgaris* against *E*. *coli* urinary isolates.

Concentrations (mg/ml)	Inhibition zone minimum–maximum (mean ± SD) in (mm)	Number of isolates showed inhibition zone (%)
12.5	8–12 (10 ± 2)	15 (28.3)
25	8–12 (10 ± 2)	29 (54.7)
50	8–15 (11.5 ± 3.5)	41 (77.4)
100	8–20 (14 ± 6)	50 (94.3)

**Table 4:** Comparison between MIC of ethanolic extract of *R. officinalis* and *T. vulgaris* against the 7 multidrug-resistant *E*. *coli* urinary isolates.

Isolate numbers	MIC (mg/ml)		MBC (mg/ml)	
	*R*. *officinalis*	*T*. *vulgaris*	*R*. *officinalis*	*T*. *vulgaris*
1	50	25	100	50
2	50	50	100	100
3	50	12.5	100	25
4	25	25	50	50
5	12.5	50	25	100
6	25	50	50	100
7	50	25	100	50

**Table 5:** DPPH Radical scavenging potential of the ethanolic extract of *R*. *officinalis* and *T*. *vulgaris*.

Sample	Rosemary	Thyme	Ascorbic acid (control +ve)
DPPH%	81.26	83.09	92

**Table 6:** Compounds, peak area%, and biological activity of *R*. *officinalis* ethanolic extract.

Name of compounds	Peak area (%)	Biological activities
Eucalyptol	18.57	Insecticide, mosquito larvicide, insect repellent, and gastroprotective activities [13]
Bicyclo [2.2.1] heptan-2-one, 1,7,7-trimethyl-, (1S)-	10.01	Antibacterial, antiallergic agents suppress various allergic inflammatory responses such as increased vascular permeability in allergic rhinitis, conjunctivitis, and asthma models [14]
Endo-borneol	4.85	Relieving symptoms of anxiety, fatigue, insomnia, anesthesia, and analgesia to alleviate abdominal pain, wounds, and burns; relieving rheumatic pain, hemorrhoids, skin diseases, and ulcers, treat cardiovascular and cerebrovascular diseases and has a significant therapeutic effect on neuralgia [15]
1,2,3,4,4a,5,6,14b-octahydrodibenz [*a*, *h*]anthracene	7.44	Antibacterial activity [16]
Flavone, 5,7-dihydroxy-8-methoxy	5.85	Anti-inflammatory activity and antihypertensive activity [17]
4,4,6a,6b,8a,11,12,14b-octamethyl-1,4,4a,5,6,6a,6b,7,8,8a,9,10,11,12,12a,14,14a,14b-octadecahydro-2H-picen-3-one	5.25	Antimicrobial activity [18]

**Table 7:** Compounds, peak area%, and biological activity of *T*. *vulgaris* ethanolic extract.

Compounds	Peak area (%)	Biological activities
Thymol	5.7	Antiseptic, antibacterial, and antifungal actions [19]
Phytol	7.92	Strong antioxidant effect and antinociceptive activity, antimicrobial, and anticancer activities [20]
*n*-hexadecanoic acid	10.27	Antibacterial activity [21]
Hexadecanoic acid, ethyl ester	18.51	Antioxidant, hemolytic, hypocholesterolemic, flavor, nematicide, and antiandrogenic [22]
Stigma sterol	2.02	Antioxidant, antimicrobial, anticancer, antiarthritic, antiasthma, anti-inflammatory, and diuretic activities [21]
Linolelaidic acid ethyl ester	17.68	Hypocholesterolemic, nematicide, antiarthritic, hepatoprotective, antiandrogenic, 5-*α* reductase inhibitor, antihistaminic, anticoronary, insectifuge, antieczemic, antiacne, and antimicrobial [23]
Ethyl 9,12,15-octadecatrienoate	14.31	Antimicrobial, anticancer, hepatoprotective, antiarthritic, antiasthma, and diuretic [21]
Gamma-sitosterol	2.59	Reduces hyperglycemia, increased insulin secretion and inhibition of glucogenesis. It can be used in the treatment of diabetes mellitus [24]

## Data Availability

The data used to support the findings of this study are available from the corresponding author upon reasonable request.
